# Abdominal, Hysterectomy for Large Fibroids, with Report of Case

**Published:** 1892-10

**Authors:** B. W Bizzell

**Affiliations:** Atlanta, Ga.


### ABDOMINAL HYSTERECTOMY FOR LARGE FIBROIDS, WITH REPORT OF CASE.

BY B. W. BIZZELL, M. D.,
ATLANTA, GA.

It is not our intention in this paper to enter into an exhaustive article upon abdominal hysterectomy,or in reporting a case to indulge in minute detail, but will deal briefly, first, with a few remarks upon uterine fibroids generally ; second, upon those requiring this form of surgical treatment. And if in relating the result of case under discussion, we are able in any wise to recommend the procedure as one justifiable, we will feel that we are at least repaid for our feeble effort.

Uterine fibroids were for a long time confounded with sarcoma, carcinoma and other malignant growths of the uterus. We also find the term tubercle used. Latterly we find such terms as fibroma and fibro-myoma.

Uterine tumors are best classified as follows:

I. Those originating from the substance proper of the uterus, varying in amount of connective tissue, or muscular tissue, in each instance.

Under this heading we include: 1. Fibroma, or fibro-myoma. As their names imply, they are reproductions of histological elements, presenting normal matured connective tissue with a varying amount of muscular tissue. 2. Sarcoma, representing embryonic connective tissue.

II. Those originating from the mucous membrane, or, perhaps more properly speaking, from the inner or glandular portion of the uterus. (For it is questionable whether we have a uterine mucous membrane.)

This class is largely composed of epithelial cells, and under this heading we make the following classification, viz.: 1, Carcinoma; 2, adenoma; 3, papilloma.

In this paper we are interested and will deal only with those growths originating from the uterine substance proper, and only go into the above classification in order to clearly understand the fibroma now under discussion.

Applied to this class of tumors we find such terms as fibroma, fibro-myoma, etc. Knowing that these tumors are composed of normal connective tissue and normal unstriped muscular tissue in varying proportions in each instance, we prefer the term fibroma.

When we speak of fibroma we simply mean a hyperplasia of normal uterine tissue, and to indicate the presence or preponderance of fibrous or connective tissue we may use the term fibroid. On the other hand, if the muscular element predominate we may use the term soft fibroid.

This distinction, from a clinical standpoint, justifies the distinctions recognized by the English writers between the white or fibrous, and the red or muscular fibroids.

The soft fibroid, the red of the English or the myoma of this country, as it is understood, is usually single and most frequently located near the fundus; while the hard fibroid, the white of the English or the fibroma of this country, are more frequently multiple and are the ones usually met with. So nearly does the substance of the soft fibroid resemble the normal uterine tissue, and so closely is it connected with the uterine wall that separation is very difficult. Microscopically a soft fibroid resembles the uterine tissue during pregnancy. The white or hard fibroid is unlike the uterine tissue in structure and is usually less firmly attached to its structure, the outer portion of which is often arranged to form a covering, or as it is called a capsule. Microscopically the white fibroid resembles ordinary fibrous or connective tissue.

Again, we have three divisions of fibroids according to point of origin: 1, Sub-mucous; 2, interstitial; 3, sub-peritoneal.

This division according to origin and situation is important, each with its peculiar symptoms accounting for the varied subjective and objective symptoms elicited during the course of fibroids. We must not forget that a once interstitial fibroid may by growth in any one direction become sub-mucous or sub-peritoneal. The symptoms vary in the three varieties; they are frequently negative in the sub-peritoneal and occasionally in the interstitial, but are usually constant in the sub-mucous.

Chief among symptoms is uterine hemorrhage or excessive menstruation; the flow is either too free, lasts too long or comes too often. Later we have derangements of neighboring organs, due to interference of nutrition from pressure. Thomas in his last work has very aptly mentioned as symptoms of fibroids a disturbance of hematomosis and the functions of the ganglionic nervous system; also mental depression and foreboding of impending death.

If we examine the mucous membrane of a fibroid uterus minutely we will find that portion underneath or covering the fibroid has become thinned and continues to grow thinner with the growth of fibroid, till finally, as says Klob, we find spaces where the utricular glands have fallen out, and according to his observation this stretching may go on till we have no mucous membrane covering that portion of uterus, but simply a mesh network. Surrounding this thinned and stretched portion of mucous membrane we have a corresponding venous engorgement and marked activity. That is, we have interstitial metritis over site of tumor and a surrounding glandular metritis, which condition invites the onset of hemorrhage characteristic of this growth of tumors.

Pain is more to be considered as the result of some accompanying complication than a symptom of the fibroid, or to mechanical pressure and nutritive changes therefrom. However, the pain is not always in proportion to size of tumor, the severest pain occurring sometimes in the smallest tumors.

While we always associate hemorrhage and occasionally pain and nervous disturbances with uterine fibroids, we must not overlook the fact that a great number of cases exist for years unobserved and are only discovered at the autopsy, no symptoms having developed during patient's life, death resulting from remote causes. In referring to causes of fibroids we are forced to admit as did Sir Charles Cloak, in 1814, "Nothing is known respecting the cause of this disease."

*Treatment.*—We should always in treating fibroids remember that they are seldom fatal within themselves, and that such a termination is the exception rather than the rule. At the same time we should not hesitate, when from any cause we believe the life of the patient is at stake, to resort to prompt surgical interference.

The treatment of fibroids as a whole is too well known and too voluminous for discussion here, and we will only take up in this instance such ones as are suited for removal per abdominal wall; those so situated as to necessitate removal of uterus and appendages also, or the operation of abdominal hysterectomy. It is with pride we note the rapid strides of the gynecologist and the successes achieved in the reported operations on the bladder, uterus, vagina and ovaries, and amid the halo of antisepsis we see his efforts crowned with success, greatest of all in abdominal hysterectomy.

At first through mistake in diagnosis and lack of courage to admit the error this operation was performed. The first successful case of this kind, where uterus and appendages were removed with fibroid, was reported by Burnham of Massachusetts (1853), followed soon after by Koeberlé (1869), with a report of nine cases with four recoveries.

In 1875 Péan, of Paris, electrified the profession by his report of numerous successful cases. Then came Hegar (1880), whose method of treating stump is still used by some.

These various operators advocated their particular mode of controlling hemorrhage and treating stump. The most successful method for controlling hemorrhage and one now generally used was the elastic tube. This was, I believe, first used by Kleeberg. While some few at that time advocated the internal treatment of stump, the majority were in favor of the external treatment, which even to-day is, we believe, best. Though should the stump be thick and short, we believe the internal method as devised by Prof. Schroeder preferable, as the tension necessary for external treatment in such cases is unwarrantable.

As before stated, the principal point at issue in the operation is the treatment of stump. Theoretically, the intra-peritoneal method is the most surgical and scientific, while the results of actual practice show the extra-peritoneal the most successful. In 153 cases of abdominal hysterectomy for fibroid tabulated and reported by Sajous in *Universal Medical Sciences* for 1889, the intra-peritoneal method was adopted in 35 cases, with 21 recoveries and 14 deaths; while the extra-peritoneal in 118 cases show 100 recoveries and 18 deaths.

*Indications for Laparotomy.*—It is generally considered good surgery to remove any ovarian tumor as soon as discovered. This, however, will not apply to fibroids. The principal indications for laparotomy are in cases of excessive hemorrhage or rapid growth and pressure on vital structures, otherwise they are not likely to prove fatal and should not be molested. Each case will, however, have to decide its own merits, recollecting that their removal is far more dangerous than ovariotomy. Large fibroids of rapid growth, causing marked systemic disturbances, are the ones especially recommended for this treatment, as ovariotomy seldom affords relief in this class.

Report of Case.—October 20, 1890, was called to see Beckie

J. (col.) and obtained the following history. Forty years old, mother of five children. Miscarried three times, the last time about five years ago. Was always rather thin, but enjoyed best of health. Menses regular, having begun at fourteen years, and appearing regularly ever since, except during her pregnancies.

About four years ago patient accidentally noticed a small tumor, or, as she expressed it, a "lump," in left inguinal region. This gave her little concern, as it was not painful and she was able to continue as a day laborer. This tumor gradually increased in size up to about eighteen months before. I saw her without any unpleasant symptoms, beyond its presence. Suddenly began to grow rapidly, and for the first time patient began to lose strength, till a few months ago she was so prostrated as to be unable to leave the house. On examination found patient very much reduced in strength and flesh. Pulse 115; respiration gave indications of pressure from large tumor pressing contents of abdominal cavity upwards. Urine normal; persistent constipation. Abdomen distended to size of pregnancy at full term, and on manipulation a symmetrical firm tumor was found, filling the entire cavity, whose attachment I seemed easily to trace to left inguinal region.

On vaginal examination found cervix high and slightly thinned. The fornices gave indications of this same firm symmetrical mass, though I thought I got decided fluctuation to left of cervix; otherwise the pelvic cavity high up seemed filled with this tumor. On account of abdominal distention could not locate and outline the uterus, which from introduction of sound seemed to be in position and of normal depth.

After repeated examination, with the history of origin in the left inguinal region, with no disturbance of menstruation and normal uterine depth, we were constrained to believe we were dealing with an ovarian tumor. Even the general appearance of patient pointed in that direction, notably wasting of upper extremities, etc. Still we recognized the fact that solid ovarian tumors of that size were very rare. As her period was sure to appear in a few days, I determined to wait results, giving laxatives and tonics. It appeared in due time, and was in every respect normal.

On account of rapid growth of tumor, with marked loss of strength, so great as to confine patient to house all the time, and a greater part to the bed, I determined to operate, and selected ten days after cessation of flow as the date for surgical interference. So, on November 7th, assisted by Dr. F. H. Bizzell, in the presence of Drs. Cheek, McWilliams and others, proceeded to operate, making incision in median line, thorough antisepsis being observed throughout.

On opening abdominal cavity I found, instead of an ovarian tumor, a large size interstitial fibroid, completely surrounding the uterus, the whole mass resembling an exaggerated uterus except low down on left side was found a lobulated mass size of hen's egg, closely adherent to large mass, but less resisting.

The incision, which extended as high as umbilicus, was insufficient to permit the exit of this solid growth, and I believed a free incision, with as little manipulation as possible, preferable to continued, and perhaps, unsuccessful efforts, with a smaller opening; the incision, therefore, was carried one and a half inches above umbilicus; a small flat sponge was first placed underneath to prevent escape of blood into cavity. Two strong silk stitches were now passed into position an inch apart at upper angle of wound; they were passed entirely through abdominal wall down, but not through, the peritoneum, then ends were secured on each side by forceps. The entire mass was then, by use of vulsellum forceps, lifted forward; a large flat sponge from sterilized solution at 110° F. was now passed back and underneath the two silk stitches, completely covering and retaining the intestines, and preventing escape of blood or foreign matter within the cavity. On right side ovary and tube was carried up and firmly united to fibroid. The ovary showed incipient cystic degeneration. Very few adhesions were found on this side. On the left side ovary and tube were found adherent to fibroid, with considerable surrounding adhesions. The ovary was found to have undergone cystic degeneration, and was the mass felt on left side. When abdomen was first opened the ovary was size of hen's egg. Adhesions were easily broken up, and the entire tumor was now removed by amputation at internal cervix. To prevent hemorrhage two steel pins were previously passed at right angles through stump, and a clamp applied below these. I intended using elastic tube, but at the last moment dropped it on the floor, so was forced to use clamp, which was easily applied, as the stump was long and thin.

Abdominal cavity was now flushed with Thiersch's solution (105° F.) and was closed in the following manner: Beginning at the lower angle the parietal peritoneum was carefully stitched to that covering stump, with cat-gut, then introducing glass drainage tube continued to approximate peritoneum with continuous cat-gut sutures. The abdominal parietes were now closed with iron dyed silk one-half inch apart. These stitches embraced all structures down to peritoneum, but not through this structure. Superficial cat-gut sutures between these completed closure. The two stitches passed at upper angle, before removing the fibroid, rendered good service by preventing escape of intestines and retaining in place the flat sponge after lifting the tumor out. The stump was now freely dusted with iodoform, and a dressing of iodoform gauze applied to incision, and wrapped around clamp and over drainage tube ; the remaining dressing was completed as usual. I prefer to simply dust the stump with iodoform to the use of cautery or acid prescribed by some.

Shock was intense, and death seemed inevitable, but with hypodermics of brandy, ether and nitro-glycerine, with free applications of heat externally, patient reacted nicely. No hemorrhage taking place the drainage tube was removed about ten hours after operation. We believe the drainage tube of no value in such cases, and will hereafter discontinue their use. Patient was able to take nourishment, developing no unpleasant symptoms till the third day, when temperature rose from 99° F. to 102° F., and patient complained of pains in bowels, accompanied with slight tympanites. Free rectal injections and moderate salines caused free movement. Next day temperature was 100° F., and no pain in bowels. The clamp was now removed from the stump, and fresh dressing applied.

On the ninth dry dressings were removed and incision found perfectly united; stitches were removed, and light dressing applied, with firm abdominal bandage. No pus formed during the reparative process except the slight sloughing of stump internally due to pressure.

An uninterrupted recovery was the result, and patient was discharged on December 20th, with instruction to keep on the abdominal bandage for some months.

It has been nearly two years now since the removal of this fibroid with ovaries and tubes intact, and it affords me much pleasure to report the woman vigorous and able to earn a living as a day laborer on a cotton plantation in Mississippi. The tumor weighed $6\frac{3}{4}$ pounds, and was a fine specimen of the fibro-myoma, and was presented to the Atlanta Society of Medicine, with short report of case, at its June meeting, 1892.

